# The Antioxidant, Analgesic, Anti-Inflammatory, and Wound Healing Activities of *Haplophyllum tuberculatum* (Forsskal) A. Juss Aqueous and Ethanolic Extract

**DOI:** 10.3390/life12101553

**Published:** 2022-10-06

**Authors:** Abdelkrim Agour, Ibrahim Mssillou, Imane Es-safi, Raffaele Conte, Hamza Mechchate, Meryem Slighoua, Fatima Ez-Zahra Amrati, Mohammad Khalid Parvez, Omer Numan, Amina Bari, Badiaa Lyoussi, Elhoussine Derwich

**Affiliations:** 1 Laboratory of Natural Substances, Pharmacology Environment, Modeling, Health and Quality of Life, Faculty of Sciences Dhar El Mahraz, University Sidi Mohamed Ben Abdellah, Fez 30000, Morocco; 2 Laboratory of Biotechnology, Health, Agrifood and Environment (LBEAS), Faculty of Sciences Dhar El Mahraz, University Sidi Mohamed Ben Abdellah, Fez 30000, Morocco; 3 Research Institute on Terrestrial Ecosystems (IRET)—CNR, 80131 Naples, Italy; 4 Department of Pharmacognosy, College of Pharmacy, King Saud University, Riyadh 11451, Saudi Arabia

**Keywords:** *H. tuberculatum*, antioxidant, wound healing, analgesic, anti-inflammatory

## Abstract

Herbal extracts are part of the solution to the increased demand for organic health care products. Traditionally, the different extracts prepared from *Haplophyllum tuberculatum* (Forsskal) A. Juss (*H. tuberculatum*) have been widely used to treat a wide range of illnesses. The aim of this study is to evaluate the antioxidant, analgesic, anti-inflammatory, and wound healing potential of the aqueous (HTAE) and ethanolic (HTEE) extracts of this plant as well as identify its major phytochemical components using LC-MS. Phytochemical analysis of both extracts revealed a rich composition and especially high amounts of glycosylic flavonols, 65.37% and 68.77% for the HTEE and HTAE, respectively. The antioxidant assays performed (DPPH, FRAP and TAC) indicated the excellent activity of the ethanolic extract while the in vivo activities (analgesic, anti-inflammatory, and healing potential) indicated the excellent activity of the aqueous extract. These findings support the therapeutic use of this plant by preventing pain and inflammation and promoting wound healing. To uncover, identify, and isolate compounds of potential medicinal and therapeutic significance, more studies on this species are required.

## 1. Introduction

Nowadays, the use of plants in the treatment of diseases is increasing. Phytomedicines can be developed based on the traditional use of plants [1]. The presence of alkaloids, coumarins, saponins, flavonoids, fixed oils, and phenolic compounds is responsible for the medicinal activity of plant species [2,3]. These natural chemical compounds can also serve society as healing, analgesic, and anti-inflammatory agents [4,5].

Among the plants used in the treatment of diseases is *Haplophyllum tuberculatum* (Forsskal) A. Juss (*H. tuberculatum*). Traditionally the juice, made from the leaves of this species, is used to treat headaches and arthritis [6]. Other extracts of *H. tuberculatum* are used in the treatment of gynecological disorders, allergic rhinitis, and asthma and also used as antispasmodic [7]. As reported in [8], other traditional uses of this species as antiseptic, vermifuge, hypnotic, calming, and wound healing, and as a treatment for ulcers, diabetes, infertility, abdominal bloating, fever, ear infections, liver diseases, rheumatism, obesity, constipation, diarrhea, colon diseases, hypertension, heart diseases, menstrual pain, and others.

*H. tuberculatum* extracts (especially the essential oil) have been the subject of several pharmacological studies. The essential oils of the aerial parts of *H. tuberculatum* have remarkable total antioxidant capacity [9] as well as anti-candida properties (against *Candida krusei*, *Candida grablata*, *Candida albicans,* and *Candida parapsilosis*) [10]. On the contrary, the essential oil was found to have a weak acetylcholinesterase inhibitory activity [11]. *H. tuberculatum* extracts also impact cardiac activity; according to [12], aqueous extract of *H. tuberculatum* decreased contractility and heart rate without affecting cardiac output in isolated and perfused rabbits, and tropine did not block the effect of aqueous extract. *H. tuberculatum* leaf extracts (petroleum ether, ethyl acetate, and butanolic extracts) could potentially reduce inflammatory and analgesic manifestations [13]. On the other hand, no study has been reported on the wound healing effect of this plant species.

Wound healing involves multiple cell populations, the extracellular matrix, and the action of soluble mediators such as growth factors and cytokines [14]. Wound healing begins with hemostasis and chemotaxis, followed by inflammation, then proliferation and finally tissue remodeling [15]. Researchers are exploring new dressings, new formulations, and new compositions of herbal medicines to develop effective, stable, and durable wound care systems [16].

*H. tuberculatum* extracts may have important pharmacological activities. In this study, we report for the first time some of the pharmacological activities of this plant, as well as the phytochemical composition of its aqueous and hydro-ethanol extract using LC/MS.

## 2. Materials and Methods

### 2.1. Preparation of the Aqueous and Hydroethanolic Extract

**Figure 1:**
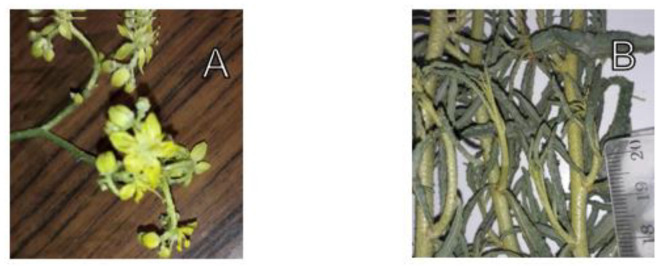
(**A**) *H. tuberculatum* flowers; (**B**) stems and leaves of *H. tuberculatum*.

The aerial parts of *H. tuberculatum* (voucher specimen: NLP02B/2021), native to southeastern Morocco (flowering stage: Figure 1) were air-dried and then ground to a fine powder with a blender (Waring^®^, Connecticut, USA). To prepare the hydro-ethanolic extract, 100 g of the powder was macerated in 1 L of 70% ethanol (Sigma-Aldrich, St. Louis, MO, USA) (700 mL of ethanol and 300 mL of distilled water). The maceration was carried out for 48 h at room temperature. The aqueous extract was prepared by infusion of 100 g of *H. tuberculatum* powder in one liter of distilled water. Both preparations were filtered through Whatman n1 paper and evaporated with a rotary evaporator (VWR, Monroeville, PA, USA). the extracts obtained were stored at a temperature of 4 °C.

### 2.2. Determination of the Phenolic Composition of Extracts by LC-MS

The analysis was conducted using a UHPLC coupled to MS/MS detector (LCMS 8060) (Shimadzu, Kyoto, Japan). Electrospray ionization was used with the MS/MS detector using the same parameters as [14]. The raw data analysis and AUC chromatograms can be found in the Supplementary Materials.

### 2.3. In Vitro Antioxidant Activity

#### 2.3.1. Determination of Total Antioxidant Capacity (TAC)

200 µL of each extract (1 mg/mL concentration) were mixed with 2 mL of the reagent solution (4 mM ammonium molybdate, 0.6 M sulfuric acid, and 28 mM sodium phosphate (Sigma-Aldrich, St. Louis, MO, USA). Then, the tubes containing the reaction solution were incubated at 95 °C for 90 min. The optical density was measured at 695 nm. Ascorbic acid was used as a standard and the total antioxidant capacity was expressed as milligram equivalents of ascorbic acid per gram of extract (Mg EAA/g extract) [17].

#### 2.3.2. Reducing Power Test

The ferric reducing power of the tested extracts was determined according to the method of [18]: 200 µL of the extract of known concentration was mixed with 500 µL of phosphate buffer (0.2 M, pH 6.6) ((Sigma-Aldrich, St. Louis, MO, USA) and 500 µL of 1% potassium ferricyanide [K_3_Fe(CN)_6_] (Sigma-Aldrich, St. Louis, MO, USA). The resulting solution was incubated at 50 °C for 20 min, the mixture was acidified with 500 µL of 10% trichloroacetic acid (TCA) (Sigma-Aldrich, St. Louis, MO, USA), and 0.5 mL of supernatant was mixed with 500 µL of distilled water and 100 µL of FeCl_3_ (0.1%). The wavelength set to measure the optic density was 700 nm, and a blank was used that contained all reagents except the extracts of *H. tuberculatum* being tested. The ascorbic acid was used as a positive control and the (EC_50_) were calculated for the expression of the results.

#### 2.3.3. Scavenging of the Free Radical DPPH

The DPPH test was performed by adopting the method of [19]. In 10 test tubes, 200 µL of different concentrations of the prepared sample were added to 1500 µL of DPPH solution previously prepared with a concentration of 4 mg/100 mL of methanol (Sigma-Aldrich, St. Louis, MO, USA). After 30 min of incubation in the dark and at room temperature, we used a spectrophotometer (VWR, Monroeville, PA, USA) to measure the optical density at 517 nm. The positive control chosen was ascorbic acid. the following equation was used to calculate the % inhibition:

(1) Inhibition %=(Ab−Aa/Ab)×100

where Ab is the optical density value of the control (−), and Aa is the optical density value of the extract.

### 2.4. Pharmacological Activities

#### 2.4.1. Animal Handling and Housing

According to [20], for the ethanolic extract of *H. tuberculatum*, the 24-h LD_50_ was approximately greater than 10 g/kg. In order to realize the analgesic, anti-inflammatory, and healing activity, male Wistar rats (120 g to 140 g) and male Swiss Albino mice (32 g to 45 g) were obtained from the animal house of the Department of Biology, Faculty of Sciences Dhar El-Mahraz, Sidi Mohamed Ben Abdellah University, Fez, Morocco under the approval of the ethical committee (July 2020/LBEAS-08 and 25 February 2021). The temperature and humidity in the animal house were 22–24 °C and 50–55%, respectively, with a day/night photoperiod (~12/12 h).

#### 2.4.2. Analgesic Activity

The analgesic effect of *H. tuberculatum* extracts was evaluated according to the method described by Karbab et al [21], using acetic acid. Thus, the mice were divided into 4 groups (5 mice in each group). In the first group (negative control), the mice received physiological water (0.9% NaCl) orally, while the second and third group received HTAE and HTEE at 500 mg/kg, respectively, in the same way. In the fourth group (positive control), the mice were treated with a well-respecified drug (Tramadol at 50 mg/kg). After 90 min of oral administration, all mice received an intraperitoneal injection of acetic acid (0.7%, *v*/*v* in saline; 10 mL/kg, i.p.). Abdominal contraction counts were performed after 5 min of acetic acid injection for 30 min. The following formula was used to calculate the percentage inhibition of abdominal contractions:

(2) PI % =((Mn−Mt)/Mn)×100

Mn: average number of abdominal contractions in the negative control group,

Mt: average number of abdominal contractions in each group treated with the extracts or standard.

#### 2.4.3. Carrageenan-Induced Rat Paw Inflammation

The anti-inflammatory potential of *H. tuberculatum* extracts (aqueous and ethanolic) was evaluated following the protocol described in the study by [22]. Wistar rats used in this test were divided into 4 groups (5 rats per group). The first group (negative control) received only physiological water (0.9% NaCl). Groups 2 and 3 were treated with both *H. tuberculatum* extracts at a dose of 500 mg/kg. Group 4, used as a standard, received indomethacin^®^ (LAPROPHAN, Casablanca, Morocco) orally (10 mg/kg). The measurement of the circumference of the right hind leg of the rats of all groups was carried out before the injection of the carrageenan suspension (1%); then the measurement was carried out again 4 times (3, 4, 5, and 6 h after the injection). Thus, we calculated the percentage of inhibition of inflammation by using the equation:

(3) PI% =((Ct−C0)Control−(Ct−C0)Treated/(Ct−C0)Control)×100

C0: The average circumference of the rat’s hind paw before injection.

Ct: The average circumference of the rat’s hind paw after carrageenan injection at a given time.

#### 2.4.4. Wound Healing Test

##### Preparation of Ointments with Extracts of H. Tuberculatum

**Figure 2:**
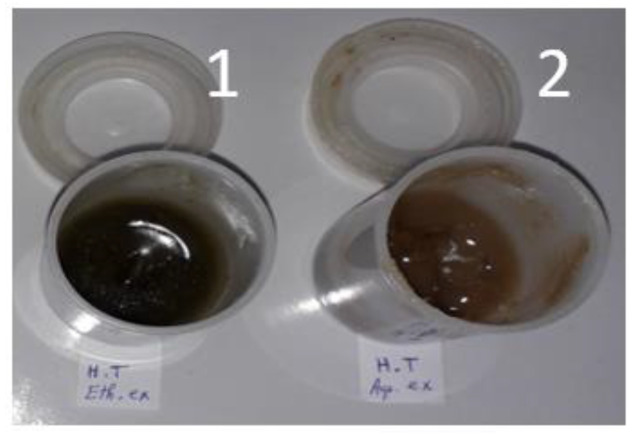
HTEE (**1**) and HTAE (**2**) extract ointment from *H. tuberculatum*.

A 10% (*w*/*w*) ointment of each extract was prepared by mixing 1 g of each extract with 9 g of Vaseline^®^. The dry extracts were gradually added to the Vaseline^®^ (LAPROPHAN, Casablanca, Morocco) in a beaker, under a temperature of 50 °C while stirring until the mixture was homogenized. Then, the extract-based ointments obtained (Figure 2) were stored at 4 °C.

#### 2.4.5. Burn Wound Induction

Induction of wounds by burning the shaved parts of rats was performed by adopting the protocol of [23]. A total of 20 rats, divided into 4 groups, were used for this test. The first group was treated with Vaseline^®^ (LAPROPHAN, Casablanca, Morocco) (negative control); the second and third groups were treated respectively with HTEE and HTAE extract ointments; and group 4 was treated with a healing ointment (Madecassol^®^ at 1%) (LAPROPHAN, Casablanca, Morocco) as standard. Anesthesia of the animals was performed intraperitoneally with pentobarbital (50 mg/kg) and the dorsal part of each rat was shaved. Burn induction, on the shaved dorsal part of each rat was performed using an aluminum rod (1.7 cm in diameter) heated in boiling water, and placed on the burn induction area for 10 s. Ointment treatment was applied daily, covering the entire wound area for 21 days. Wound surfaces were photographed with a digital camera in the presence of a graduated ruler (scale). The photographs obtained were analyzed by ImageJ software. In order to calculate the burn-induced wound contraction rate, the following formula was used:

(4) WC%=((WA0−WAGD)/WA0)×100

WC %: % of wound contraction

WA0: Wound area after day 1

WAGD: Wound area on a given day

### 2.5. Statistical Analysis

GraphPad Prism version 8 software (San Diego, CA, USA, accessed date 1 July 2021) was used to process the results, statistical differences were assessed by one-way ANOVA test. A value of *p* < 0.05 was considered statistically significant. All results were expressed as mean ± SD.

## 3. Results and Discussion

### 3.1. Phenolic Composition of HTEE and HTAE Extracts by LC-MS

LC-MS analysis revealed a total phenolic content of 99.38 percent (17 compounds) and 99.77 percent (9 compounds) in the HTEE extract and HTAE extract of *H. tuberculatum*, respectively (Table 1). Both extracts had a higher percentage of glycosidic flavonols, with values higher than 65% (Table 2). Moreover, the ethanolic extract contained a high amount of flavanols (22.98%), and 7.25% of phenolic acids. While the aqueous extract contained a large amount of condensed tannins (18.75% procyanidins) and 7.28% flavonols. The aqueous extract differed from the ethanolic extract by the presence of flavanone (4.97% naringin).

In previous studies, different solvents have been used to obtain extracts from *H. tuberculatum*, and several techniques were adopted to separate, isolate, or identify the extracted molecules. Among the phenolic compounds mentioned are coumarins, flavonoids, and lignans. Ammoidin is an example of coumarins isolated from petroleum ether extract and determined by HPLC–UV [24], the flavonoid 5,7,4′-trihydroxy-6-methoxy-3-*O*-glucosyl, a flavone, was identified in ethyl acetate extract by CC, IR, UV, NMR, and MS [25]. In the group of lignans, [26] identified γ-lactone and (-)-secoisolariciresinol in a methanolic extract of the whole plant. Likewise, [27,28] isolated several molecules from this group, including acetyl-tuberculatin, justicidin A, justicidin B, and tuberculatin. Diphyllin is also a lignan identified by [25]. *H. tuberculatum* is a species that also contains fatty acids; a chromatographic analysis (GC-FID) revealed that the petroleum ether extract contains γ-linolenic acid, palmitic acid, linoleic acid, erucic acid, and stearic acid in percentages of 45.50%, 18.48%, 10.73%, 4.72%, and 3.96%, respectively [13].

The phytochemicals found in *H. tuberculatum* vary, mostly depending on the plant material employed, extraction solvents, and analytical procedures. However, [29] reported that ethanol was very effective as an extraction solvent for different classes of polar and non-polar compounds, while methanol, dichloromethane, n-hexane, chloroform, petroleum ether, and ethyl acetate can be effective in the extraction of compounds such as lignans, coumarins, and alkaloids.

### 3.2. Antioxidant Activity (TAC, DPPH an FRAP Tests)

The results of antioxidant activity are represented in Table 3. The TAC test revealed that the HTEE extract yielded a high total antioxidant capacity, with a value of 280.98 ± 5.32 mg AAE/g extract, while the aqueous extract yielded low antioxidant activity of 85.41 ± 1.33 mg AAE/g extract. HTEE was active against DPPH radical, with an IC_50_ value of 0.371 mg/mL, a higher value compared to that obtained using ascorbic acid (IC_50_ = 0.0027 mg/mL). HTAE remained almost inactive against DPPH. On the other hand, the FRAP test revealed that HTAE had a higher iron reduction capacity than HTEE. In general, antioxidant activity tests revealed that HTEE had significant antioxidant activity compared to HTAE. These results can be explained by the richness of HTEE in polyphenolic molecules with high antioxidant activity.

In the bibliography, the authors of [30] reported that the IC_50_ (mg/mL) of aqueous extract of *H. tuberculatum* leaves were 0.447, 0.914, and 0.463, respectively for DPPH, ABTS, and β-carotene, while the values obtained by methanolic extract were 0.758, 0.29, and 0.457, respectively for DPPH, ABTS, and β-carotene. The authors of [31] reported that the ethanolic extract exhibited an antiradical activity (ORAC assay) of approximately 1.283 µmol TE/mg of sample. In addition, this extract inhibited intracellular ROS production with an IC_50_ of 0.026 µg/mL. The antioxidant activity of the ethanolic extract of *H. tuberculatum* aerial parts was examined by determination of glutathione in the blood of alloxan-induced diabetic rats; this extract showed a great antioxidant activity compared to vitamin E [20]. On the other hand, methanolic extract has DPPH-scavenging capacity, with an IC_50_ value = 40.33 µg/mL [32]. Other extracts obtained from *H. tuberculatum* leaves showed significant total antioxidant capacity equivalent to that of ascorbic acid. However, free radical scavenging activity (DPPH) was slightly lower than that of the reference compound [33].

### 3.3. Pharmacological Activities

#### 3.3.1. Peripheral Analgesic Activity

**Figure 3:**
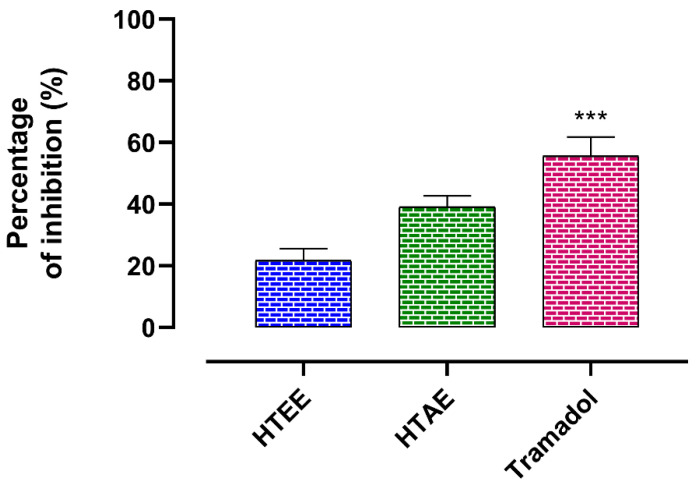
Analgesic activity of *H. tuberculatum* extracts and the standard compound tramadol. Data are expressed as means ± SEM (*n* = 5). *** *p* < 0.001 compared to both plant extracts.

The results of the analgesic activity are presented in Figure 3. The HTAE exerted a remarkable analgesic activity with a percentage of inhibition of 39.15 ± 5.70%, while the HTEE exerted a moderate activity with a % of inhibition of 21.68 ± 7.22%. HTEE also shows low analgesic activity compared to tramadol (55.64 ± 12.04%).

Few works have reported the analgesic effect of extracts obtained from *H. tuberculatum*. Comparing the results obtained with those found by [28], chloroform (100 mg/kg), ethyl acetate (50 mg/kg), butanol (100 mg/kg), and petroleum ether (100 mg/kg) extracts of *H. tuberculatum* exhibited peripheral analgesic activity using the acetic acid constriction test in mice; the percentages of inhibition were 46.42%, 75.23%, 75.78%, and 69.28%, respectively. In the same study, *H. tuberculatum* extracts exerted central analgesic activity, tested by hot-plate test in mice; thus, butanolic (100 mg/kg) and ethyl acetate (50 mg/kg) petroleum ether (100 mg/kg) extracts had similar analgesic activities compared to the standard used, lysine acetylsalicylate, after 30, 60, and 90 min of response latency.

#### 3.3.2. Anti-Inflammatory Activity

**Figure 4:**
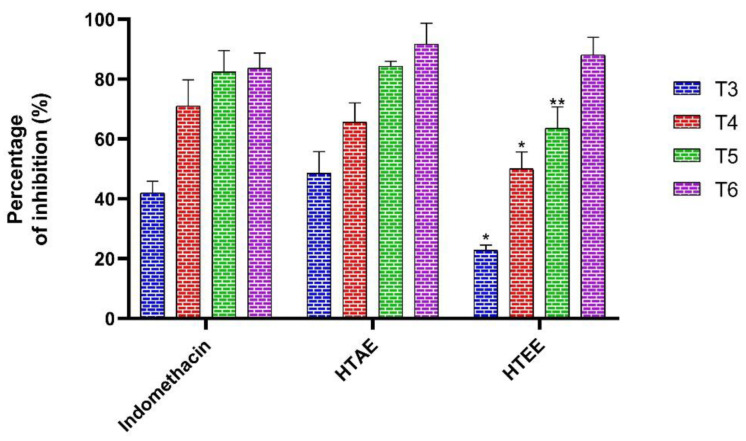
Anti-inflammatory activity of *H. tuberculatum* extracts and the standard compound. (T3: 3rd hour; T4: 4th hour; T5: 5th hour and T6; 6th hour); Data are expressed as mean as means ± SEM (*n* = 5). * *p* < 0.05 and ** *p* < 0.01 compared to Indomethacin.

The results obtained in the anti-inflammatory test are shown in Figure 4. It can be seen that the anti-inflammatory activity of the studied extracts is comparable to that obtained using indomethacin (89%). The richness in different phenolic compounds of both extracts boosted the anti-inflammatory activity potential as it increases gradually with time; its highest % inhibition was recorded during the 6th hour (90% HTAE and 85% HTEE, respectively).

For analgesic activity, until now, few works have reported the anti-inflammatory effect of extracts obtained from *H. tuberculatum.* According to [28], at doses of 25, 50, and 100 mg/kg, petroleum ether extract exhibited inhibition percentages of 51.12, 86.71, and 96.92%, respectively, 5 h after carrageenan injection. In [20] it was reported that the percentage of edema inhibition (induced by carrageenan in rats) of the flower essential oil and ethanolic extracts of *H. tuberculatum* aerial parts were 8.56%, 9.52%, and 10.43%. 

#### 3.3.3. Wound Healing Activity of *H. tuberculatum* Extracts

**Figure 5:**
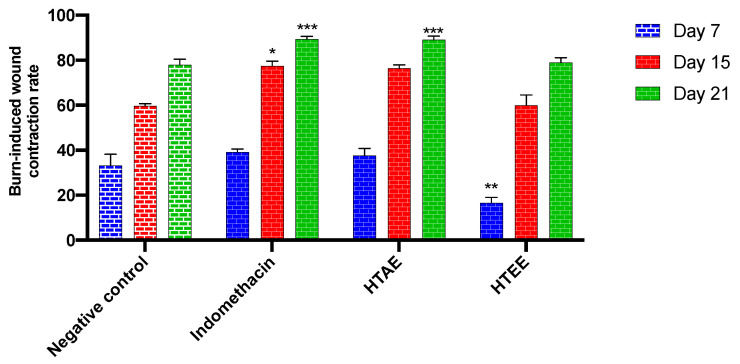
Percentage of wound contraction by using *H. tuberculatum* extracts and indomethacin (standard). Data are expressed as means ± SEM (*n* = 4). * *p* < 0.05, ** *p* < 0.01, and *** *p* < 0.001 compared to negative control.

**Figure 6:**
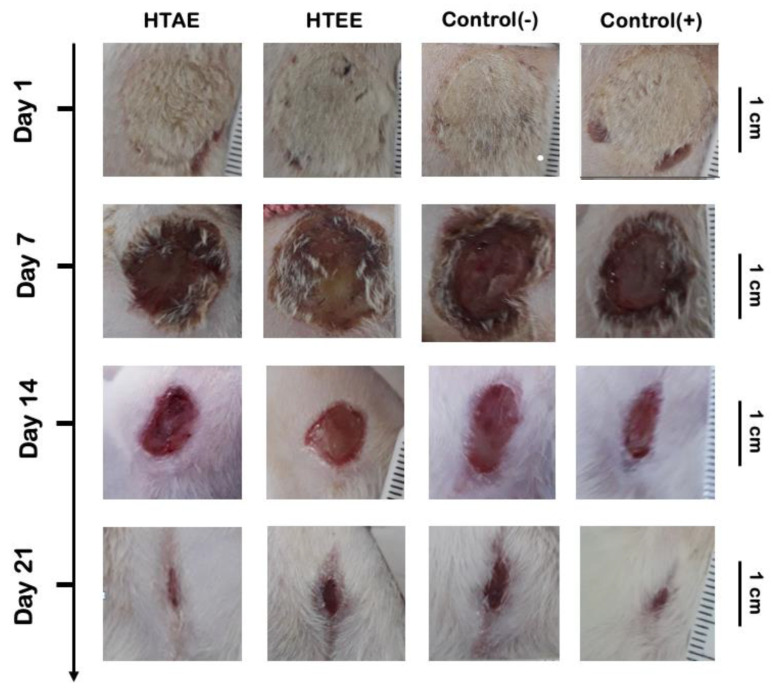
Images of the healing process in treated rats.

The wound healing ability of *H. tuberculatum* extracts was examined in Wistar rats. Differences in the rate of wound healing as a function of time in the groups treated for 3 weeks are presented in Figure 5, and the images of the wound healing process are shown in Figure 6. During the 3 weeks of treatment, the rats showed no signs of extract-related toxicity or other skin-sensitivity side effects. Contraction of the burn wound was observed from the 6th day.

The results obtained show significant differences in the wound healing rate between the four groups as early as day 7 after the beginning of the treatment. However, the wound healing rates of the positive control and HTAE groups were significantly higher than those of the negative control, Vaseline-treated (78%), and HTEE groups 79%. At day 21, the healing rates of the HTAE and positive control groups were greater than 89%. This is the first study to report the healing activity of *H. tuberculatum* extracts. According to the results obtained, and those found in the bibliography, we can suggest that the composition of the aqueous extract of *H. tuberculatum* in polyphenols, especially flavonoids, gives it a healing potential [34,35].

## 4. Conclusions

From the results of this study, we can say that the hydro-ethanolic extract of *H. tuberculatum* (HTEE) can be strongly recommended as an antioxidant product, due to its high composition of phenolic compounds, as revealed by chromatographic analysis (LC-MS). On the other hand, the aqueous extract of *H. tuberculatum* (HTAE) can form the basis of a phytomedicine with healing activity, due to its analgesic, anti-inflammatory, and healing powers realized by in vivo tests. In addition, more detailed studies on the isolation of *H. tuberculatum* compounds, as well as their pharmacological studies, are strongly suggested in order to discover molecules of clinical utility.

## Figures and Tables

**Table 1:** Phenolic composition by LC-MS of HTEE and HTAE of *H. tuberculatum*.

	HTEE		HTAE	
	AUC	%	AUC	%
***p*-Coumaric acid**	989,312	0.38%	-	-
**Quercetin-3-*O*-glucoside**	9,957,638	3.83%	-	-
**Quercetin-3-*O*-glucuronic acid**	32,205,427	12.04%	4,165,106	4.18%
**Kaempferol-3-*O*-glucose**	41,966,154	16.16%	6,337,995	6.37%
**Quercetin-3-*O*-hexose deoxyhexose**	41,926,648	16.14%	6,214,174	6.24%
**Isorhamnetin-7-*O*-pentose**	10,513,919	4.05%	-	-
**Luteolin 7-*O*-glucoside**	10,652,328	4.05%	-	-
**Kaempferol-3-*O*-glucuronic acid**	10,988,309	4.05%	2,887,322	2.90%
**Kaempferol-3-*O*-hexose deoxyhexose**	13,135,204	5.05%	48,833,492	49.08%
**Protocatechuic acid**	5,194,517	2.00%	190,045	0.19%
***p*-Hydroxybenzoic\Salicylic acid**	1,105,533	0.42%	-	-
**Gentisic acid**	5,324,786	2.04%	-	-
**Synaptic acid**	1,977,146	0.76%	-	-
**Ferulic acid**	4,290,764	1.65%	-	-
**Gallocatechin\Epigallocatechin**	12,874,992	4.96%	-	-
**Procyanidins**	98,17,388	3.78%	18,653,758	18.75%
**Rutin**	46,831,385	18.02%	7,251,975	7.28%
**Naringin**	-	-	4,945,206	4.97%
**Total**	259,751,450	100%	99,479,073	100%

**Table 2:** Percentages of different phenolic types in *H. tuberculatum* extracts.

	HTEE	HTAE
**Flavonol glycosides**	65.37%	68.77%
**Flavanols**	22.98%	7.28%
**Flavanone**	-	4.97%
**Condensed tannin**	3.78%	18.75%
**Phenolic acids**	7.25%	0.19%

**Table 3:** Antioxidant activity of *H. tuberculatum* extracts.

DPPH (IC_50_ mg/mL)			FRAP (EC_50_ mg/mL)			TAC (mg AAE/g Extract)	
HTEE	HTAE	A.As	HTEE	HTAE	A.As	HTEE	HTAE
**0.37104**	-	0.00279	0.16944	0.15554	0.00297	280.98 ± 5.32	85.41 ± 1.33

## Data Availability

Data are available upon request.

## Supplementary Materials

The following supporting information can be downloaded at: www.mdpi.com/article/10.3390/life12101553/s1, Figure S1: HTAE LC/MS DATA; Figure S2: HTEE LC/MS DATA.
