# Supplementary material for: The Antioxidant, Analgesic, Anti-Inflammatory, and Wound Healing Activities of Haplophyllum tuberculatum (Forsskal) A. Juss Aqueous and Ethanolic Extract
Source: Life (Basel). 2022 Oct 6;12(10):1553. doi: 10.3390/life12101553 (PMC9605159; doi:10.3390/life12101553)
Supplement: Supplementary file 1 [file life-12-01553-s001.zip › HTEE LC:MS DATA.pdf]

# = Shimadzu LabSolutions Quant. Browser Data Report =

Acquired by	: System Administrator
Data Acquired	: 10/12/2021 11:59:18
Sample Type	: Unknown
Sample Name	: 10
Sample ID	:
Sample Amount	: 1
Dilution Factor	: 1
Vial#	: 57
Injection Volume	: 10 uL
Data Filename	: 10_013.lcd
Method Filename	: polifenoli screening SIM.lcm
Processed by	: System Administrator
Modified Date	: 10/12/2021 12:00:02

198.9000(+)@1ID#:1 m/z: 198.9000

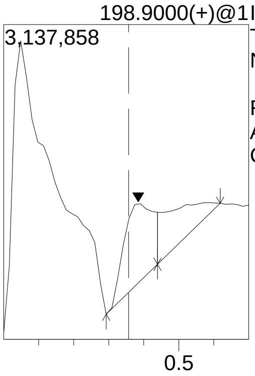

Type:Target
Name:syringic acid SIM

Ret.Time:0.384
Area:4575343
Conc.:0.000ppm

168.9000(-)@2ID#:2 m/z: 168.9000

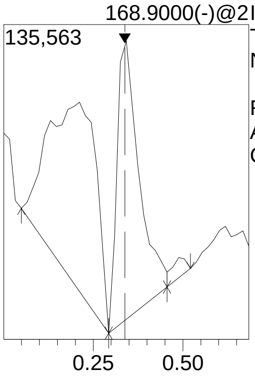

Type:Target
Name:gallic acid SIM

Ret.Time:0.338
Area:357841
Conc.:0.000ppm

301.0000(-)@3ID#:3 m/z: 301.0000

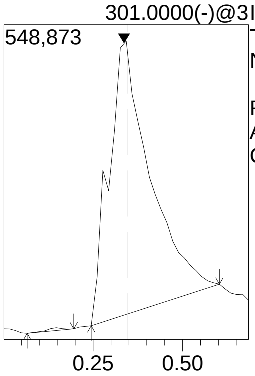

Type:Target
Name:quercetin sim

Ret.Time:0.336
Area:3731516
Conc.:0.000ppm

162.9000(-)@4ID#:4 m/z: 162.9000

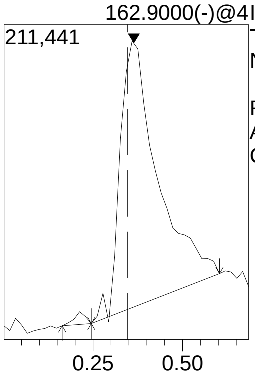

Type:Target
Name:p coumarci acid SIM

Ret.Time:0.364
Area:1011122
Conc.:0.000ppm

303.2000(-)@5ID#:5 m/z: 303.2000

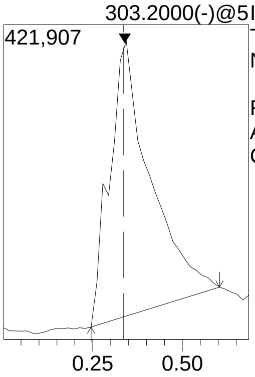

Type:Target
Name:oleochantal SIM

Ret.Time:0.340
Area:2806346
Conc.:0.000ppm

153.0500(-)@6ID#:6 m/z: 153.0500

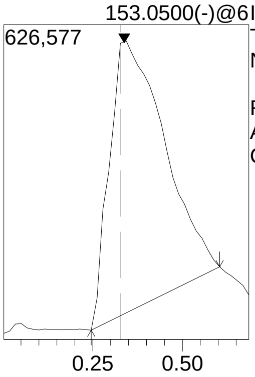

Type:Target
Name:hydroxytyrosol SIM

Ret.Time:0.338
Area:5660398
Conc.:0.000ppm

193.0000(-)@7ID#:7 m/z: 193.0000

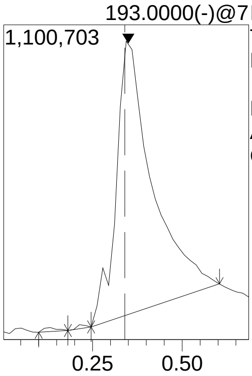

Type:Target
Name:trans ferulic acid SIM

Ret.Time:0.350
Area:6097387
Conc.:0.000ppm

539.0000(-)@8ID#:8 m/z: 539.0000

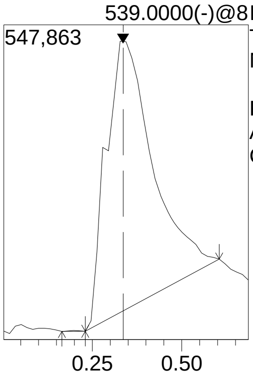

Type:Target
Name:oleuropein SIM

Ret.Time:0.336
Area:4167918
Conc.:0.000ppm

301.3000(-)@9ID#:9 m/z: 301.3000

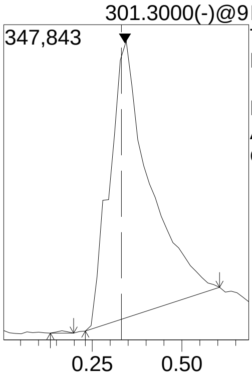

Type:Target
Name:Hesperetin

Ret.Time:0.341
Area:2304117
Conc.:0.000ppm

312.0000(-)@10ID#:10 m/z: 312.0000

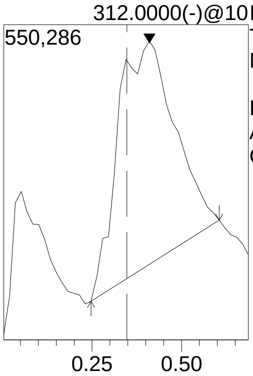

Type:Target
Name:trimethoxyflavone

Ret.Time:0.410
Area:4070805
Conc.:0.000ppm

271.2000(-)@11ID#:11 m/z: 271.2000

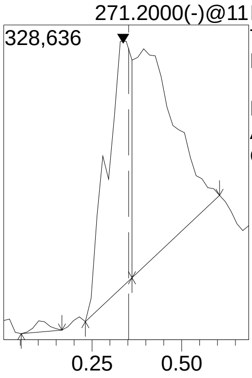

Type:Target
Name:Arbutin

Ret.Time:0.337
Area:951549
Conc.:0.000ppm

359.0000(-)@12ID#:12 m/z: 359.0000

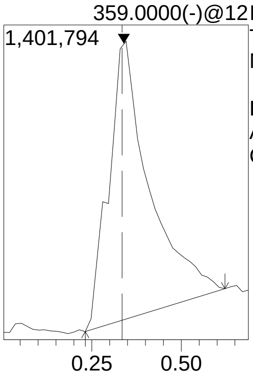

Type:Target
Name:Rosmarinic acid

Ret.Time:0.340
Area:9537230
Conc.:0.000ppm

455.0000(-)@13ID#:13 m/z: 455.0000

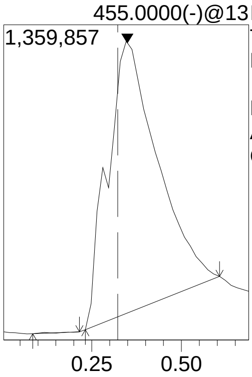

Type:Target
Name:ursolic acid

Ret.Time:0.349
Area:11132523
Conc.:0.000ppm

269.0000(-)@14ID#:14 m/z: 269.0000

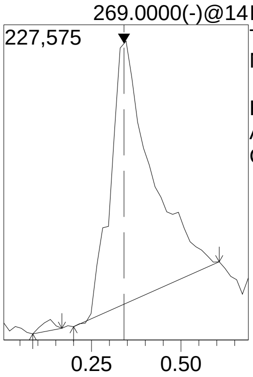

Type:Target
Name:Apigenin

Ret.Time:0.341
Area:1284773
Conc.:0.000ppm

537.1000(-)@15ID#:15 m/z: 537.1000

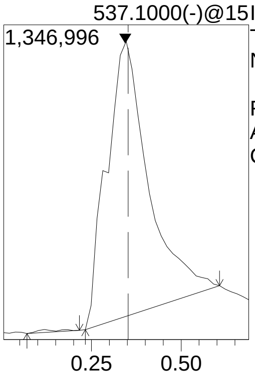

Type:Target
Name:Amentoflavone

Ret.Time:0.344
Area:9329209
Conc.:0.000ppm

284.9000(-)@16ID#:16 m/z: 284.9000

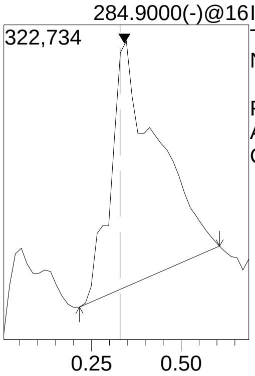

Type:Target
Name:luteoilin

Ret.Time:0.342
Area:1940556
Conc.:0.000ppm

463.1000(-)@17ID#:17 m/z: 463.1000

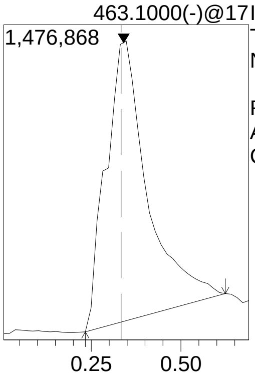

Type:Target
Name:quercetin-3-O-glucoside

Ret.Time:0.341
Area:10435736
Conc.:0.000ppm

477.0000(-)@18ID#:18 m/z: 477.0000

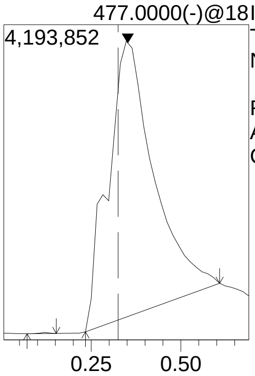

Type:Target
Name:quercetin-3-O- glucuronic acid

Ret.Time:0.352
Area:32541960
Conc.:0.000ppm

609.1000(-)@19ID#:19 m/z: 609.1000

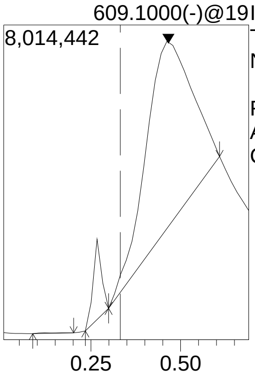

Type:Target
Name:Kaempferol-3-O-glucose

Ret.Time:0.466
Area:42270641
Conc.:0.000ppm

609.1000(-)@20ID#:20 m/z: 609.1000

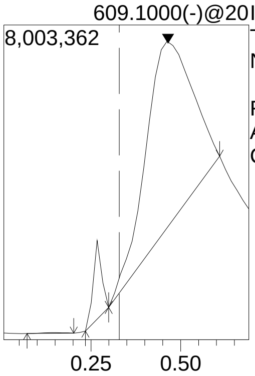

Type:Target
Name:Quercetin-3-O.hexose deoxyhex

Ret.Time:0.465
Area:42262841
Conc.:0.000ppm

623.1000(-)@21ID#:21 m/z: 623.1000

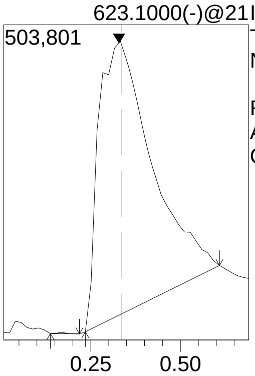

Type:Target
Name:Isorhamnetin- 3-O Rutinoside

Ret.Time:0.329
Area:4372012
Conc.:0.000ppm

447.1000(-)@22ID#:22 m/z: 447.1000

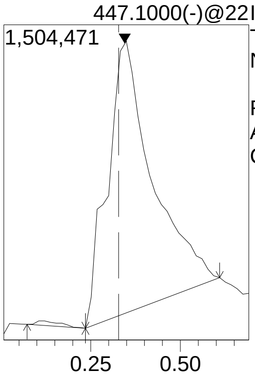

Type:Target
Name:Isorhamnetin-7-O- Pentose

Ret.Time:0.345
Area:10683811
Conc.:0.000ppm

447.1000(-)@23ID#:23 m/z: 447.1000

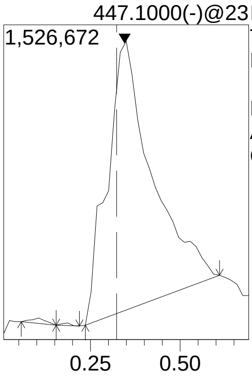

Type:Target
Name:luteoilin 7-O-glucoside

Ret.Time:0.345
Area:10807795
Conc.:0.000ppm

461.1000(-)@24ID#:24 m/z: 461.1000

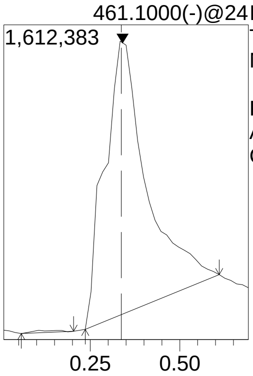

Type:Target
Name:Kaempferol-3-O-glucuronic acid

Ret.Time:0.340
Area:11215892
Conc.:0.000ppm

417.1000(-)@25ID#:25 m/z: 417.1000

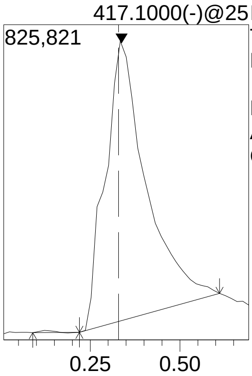

Type:Target
Name:Kaempferol-3-O-pentose

Ret.Time:0.337
Area:5681988
Conc.:0.000ppm

593.1000(-)@26ID#:26 m/z: 593.1000

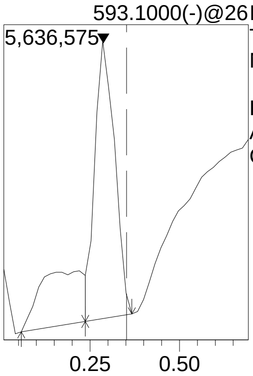

Type:Target
Name:Kaempferol-3-O-hexose deohyhe

Ret.Time:0.287
Area:13831592
Conc.:0.000ppm

153.4000(-)@27ID#:27 m/z: 153.4000

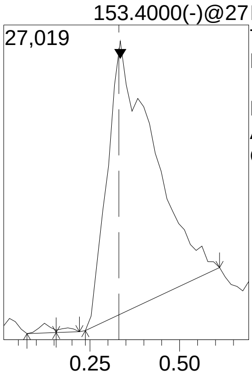

Type:Target
Name:Tyrosol

Ret.Time:0.335
Area:204248
Conc.:0.000ppm

153.0000(-)@28ID#:28 m/z: 153.0000

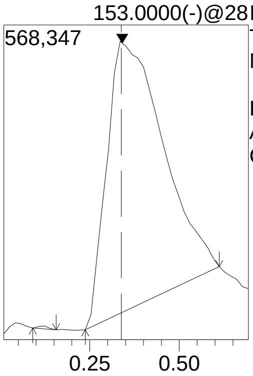

Type:Target
Name:Protocathecoic acid

Ret.Time:0.341
Area:5238135
Conc.:0.000ppm

167.0000(-)@29ID#:29 m/z: 167.0000

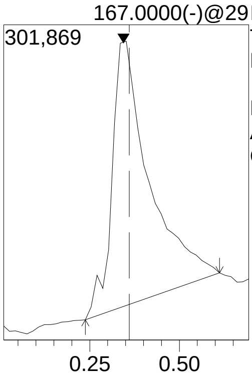

Type:Target
Name:Vanillic acid

Ret.Time:0.344
Area:1676976
Conc.:0.000ppm

197.0000(-)@30ID#:30 m/z: 197.0000

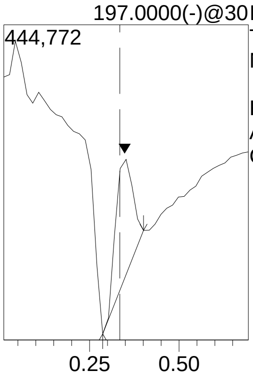

Type:Target
Name:syringic acid

Ret.Time:0.348
Area:456648
Conc.:0.000ppm

137.0000(-)@31ID#:31 m/z: 137.0000

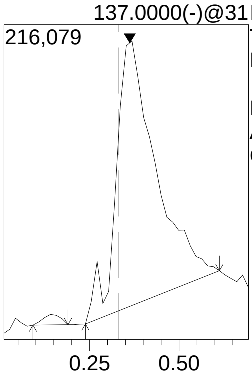

Type:Target
Name:p-hydroxybenzoic\salicilic acid

Ret.Time:0.362
Area:1175717
Conc.:0.000ppm

153.0000(-)@32ID#:32 m/z: 153.0000

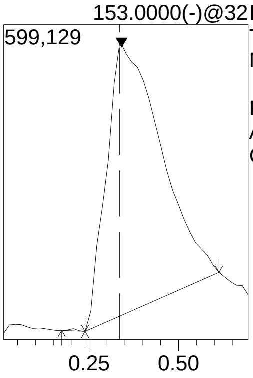

Type:Target
Name:Gentisic acid

Ret.Time:0.341
Area:5362697
Conc.:0.000ppm

179.0000(-)@33ID#:33 m/z: 179.0000

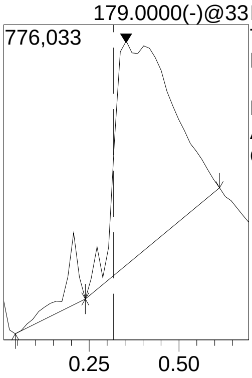

Type:Target
Name:Caffeic acid

Ret.Time:0.353
Area:5001791
Conc.:0.000ppm

223.0000(-)@34ID#:34 m/z: 223.0000

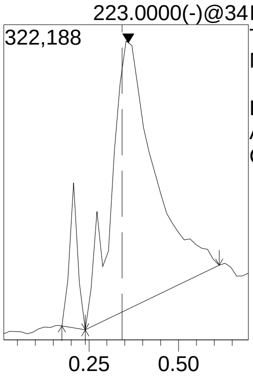

Type:Target
Name:Sinapic acid

Ret.Time:0.360
Area:2069373
Conc.:0.000ppm

193.0000(-)@35ID#:35 m/z: 193.0000

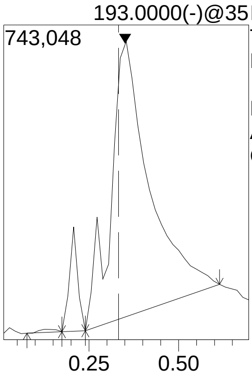

Type:Target
Name:Ferulic acid

Ret.Time:0.351
Area:4386403
Conc.:0.000ppm

147.0000(-)@36ID#:36 m/z: 147.0000

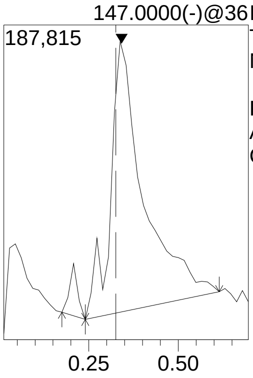

Type:Target
Name:Trans-cinnamic acid

Ret.Time:0.342
Area:924994
Conc.:0.000ppm

353.0000(-)@37ID#:37 m/z: 353.0000

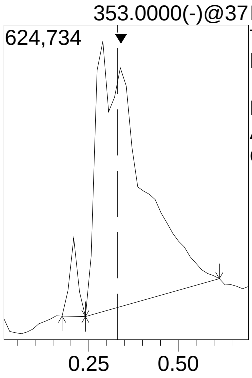

Type:Target
Name:Chlorogenic acid

Ret.Time:0.340
Area:3917422
Conc.:0.000ppm

289.0000(-)@38ID#:38 m/z: 289.0000

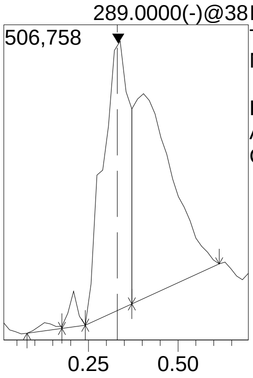

Type:Target
Name:cathechin\epicathechin

Ret.Time:0.333
Area:1969756
Conc.:0.000ppm

457.0000(-)@39ID#:39 m/z: 457.0000

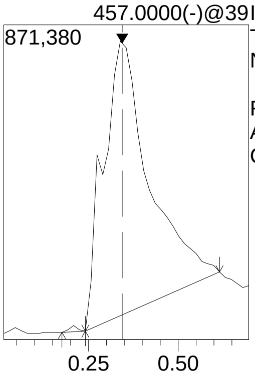

Type:Target
Name:gallocathechin\epigallocathechin

Ret.Time:0.344
Area:6576975
Conc.:0.000ppm

305.0000(-)@40ID#:40 m/z: 305.0000

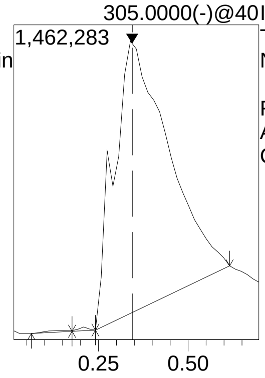

Type:Target
Name:gallocathechin\epigallocathechin

Ret.Time:0.344
Area:13038404
Conc.:0.000ppm

441.0000(-)@41ID#:41 m/z: 441.0000

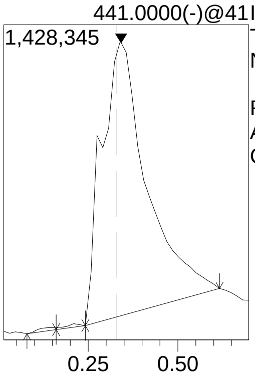

Type:Target
Name:cathechin gallate

Ret.Time:0.342
Area:10329434
Conc.:0.000ppm

577.0000(-)@42ID#:42 m/z: 577.0000

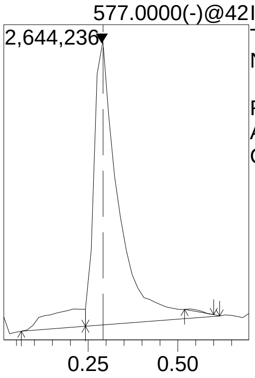

Type:Target
Name:Procianidin

Ret.Time:0.288
Area:10074849
Conc.:0.000ppm

317.0000(-)@43ID#:43 m/z: 317.0000

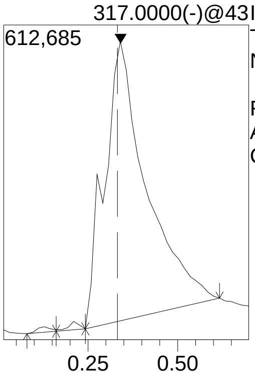

Type:Target
Name:Myricetin

Ret.Time:0.341
Area:4197266
Conc.:0.000ppm

285.0000(-)@44ID#:44 m/z: 285.0000

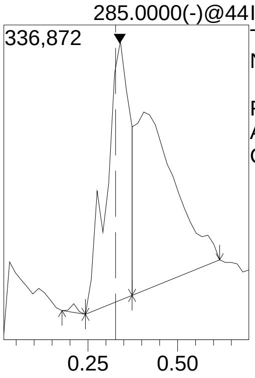

Type:Target
Name:Kaempeferol

Ret.Time:0.339
Area:984569
Conc.:0.000ppm

609.0000(-)@45ID#:45 m/z: 609.0000

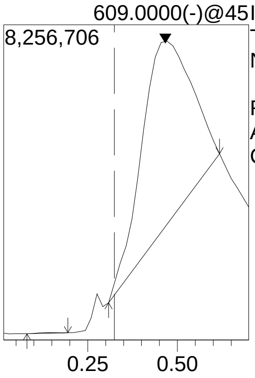

Type:Target
Name:rutin

Ret.Time:0.467
Area:47135081
Conc.:0.000ppm

579.0000(-)@46ID#:46 m/z: 579.0000

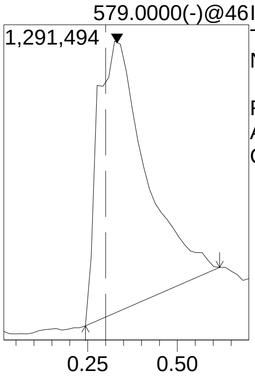

Type:Target
Name:narigin

Ret.Time:0.332
Area:9885573
Conc.:0.000ppm
