# Supplementary material for: The Antioxidant, Analgesic, Anti-Inflammatory, and Wound Healing Activities of Haplophyllum tuberculatum (Forsskal) A. Juss Aqueous and Ethanolic Extract
Source: Life (Basel). 2022 Oct 6;12(10):1553. doi: 10.3390/life12101553 (PMC9605159; doi:10.3390/life12101553)
Supplement: Supplementary file 1 [file life-12-01553-s001.zip › HTAE LC:MS DATA.pdf]

# = Shimadzu LabSolutions Quant. Browser Data Report =

Acquired by	: System Administrator
Data Acquired	: 10/12/2021 12:01:01
Sample Type	: Unknown
Sample Name	: 11
Sample ID	:
Sample Amount	: 1
Dilution Factor	: 1
Vial#	: 58
Injection Volume	: 10 uL
Data Filename	: 11_014.lcd
Method Filename	: polifenoli screening SIM.lcm
Processed by	: System Administrator
Modified Date	: 10/12/2021 12:01:45

198.9000(+)@1ID#:1 m/z: 198.9000

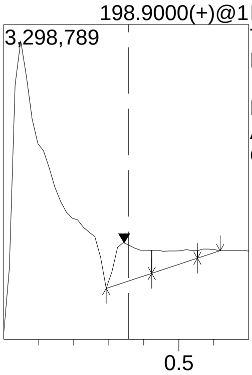

Type:Target
Name:syringic acid SIM

Ret.Time:0.344
Area:2322271
Conc.:0.000ppm

168.9000(-)@2ID#:2 m/z: 168.9000

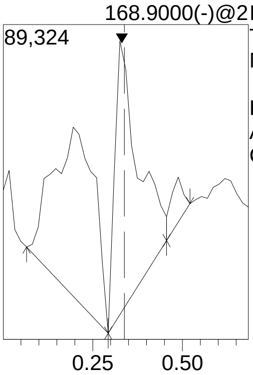

Type:Target
Name:gallic acid SIM

Ret.Time:0.331
Area:242543
Conc.:0.000ppm

301.0000(-)@3ID#:3 m/z: 301.0000

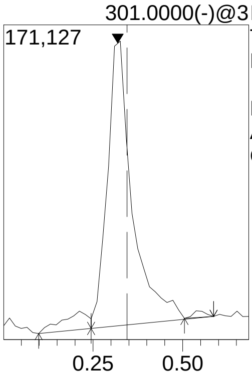

Type:Target
Name:quercetin sim

Ret.Time:0.319
Area:640735
Conc.:0.000ppm

162.9000(-)@4ID#:4 m/z: 162.9000

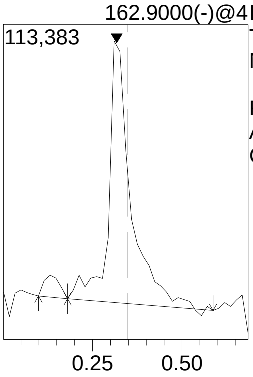

Type:Target
Name:p coumarci acid SIM

Ret.Time:0.318
Area:293225
Conc.:0.000ppm

303.2000(-)@5ID#:5 m/z: 303.2000

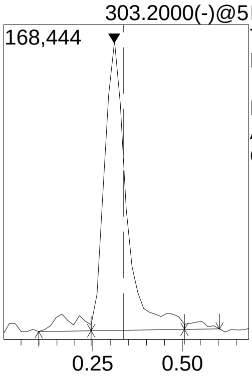

Type:Target
Name:oleochantal SIM

Ret.Time:0.310
Area:568743
Conc.:0.000ppm

153.0500(-)@6ID#:6 m/z: 153.0500

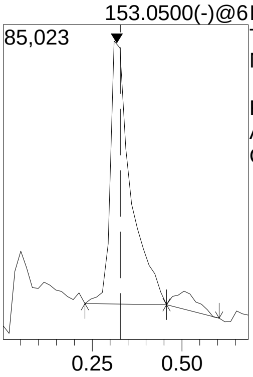

Type:Target
Name:hydroxytyrosol SIM

Ret.Time:0.319
Area:223184
Conc.:0.000ppm

193.0000(-)@7ID#:7 m/z: 193.0000

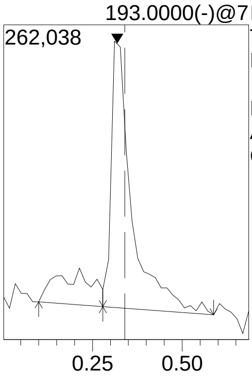

Type:Target
Name:trans ferulic acid SIM

Ret.Time:0.319
Area:594356
Conc.:0.000ppm

539.0000(-)@8ID#:8 m/z: 539.0000

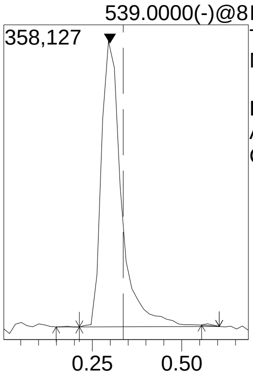

Type:Target
Name:oleuropein SIM

Ret.Time:0.299
Area:1219206
Conc.:0.000ppm

301.3000(-)@9ID#:9 m/z: 301.3000

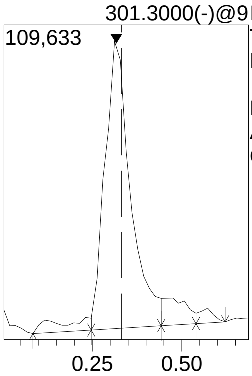

Type:Target
Name:Hesperetin

Ret.Time:0.317
Area:421870
Conc.:0.000ppm

312.0000(-)@10ID#:10 m/z: 312.0000

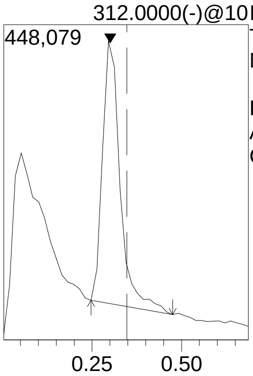

Type:Target
Name:trimethoxyflavone

Ret.Time:0.301
Area:1119817
Conc.:0.000ppm

271.2000(-)@11ID#:11 m/z: 271.2000

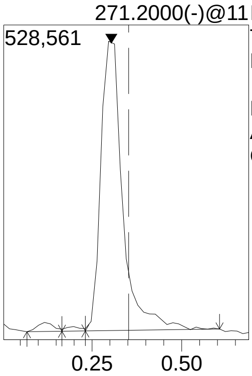

Type:Target
Name:Arbutin

Ret.Time:0.304
Area:1832073
Conc.:0.000ppm

359.0000(-)@12ID#:12 m/z: 359.0000

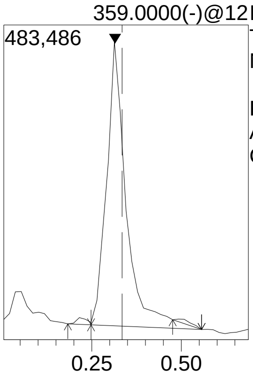

Type:Target
Name:Rosmarinic acid

Ret.Time:0.315
Area:1454287
Conc.:0.000ppm

455.0000(-)@13ID#:13 m/z: 455.0000

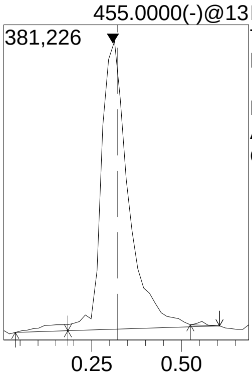

Type:Target
Name:ursolic acid

Ret.Time:0.309
Area:1604857
Conc.:0.000ppm

269.0000(-)@14ID#:14 m/z: 269.0000

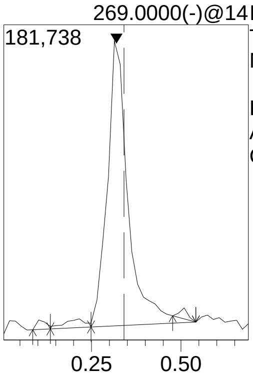

Type:Target
Name:Apigenin

Ret.Time:0.319
Area:536988
Conc.:0.000ppm

537.1000(-)@15ID#:15 m/z: 537.1000

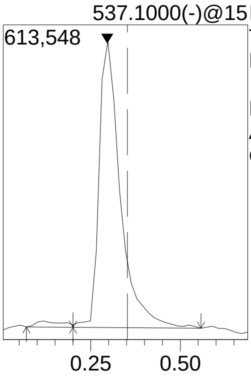

Type:Target
Name:Amentoflavone

Ret.Time:0.296
Area:2174141
Conc.:0.000ppm

284.9000(-)@16ID#:16 m/z: 284.9000

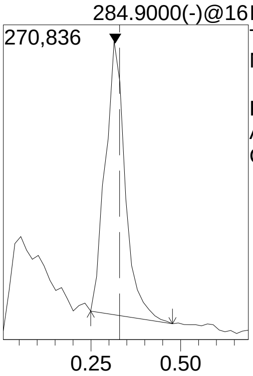

Type:Target
Name:luteoilin

Ret.Time:0.318
Area:728438
Conc.:0.000ppm

463.1000(-)@17ID#:17 m/z: 463.1000

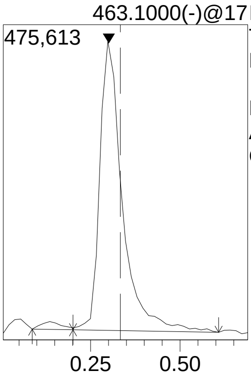

Type:Target
Name:quercetin-3-O-glucoside

Ret.Time:0.301
Area:1754910
Conc.:0.000ppm

477.0000(-)@18ID#:18 m/z: 477.0000

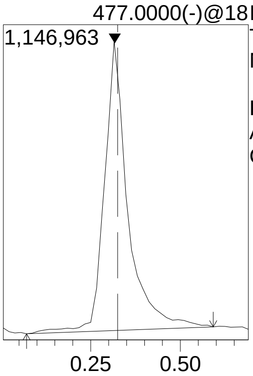

Type:Target
Name:quercetin-3-O- glucuronic acid

Ret.Time:0.317
Area:4501639
Conc.:0.000ppm

609.1000(-)@19ID#:19 m/z: 609.1000

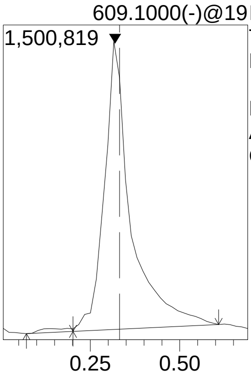

Type:Target
Name:Kaempferol-3-O-glucose

Ret.Time:0.319
Area:6642482
Conc.:0.000ppm

609.1000(-)@20ID#:20 m/z: 609.1000

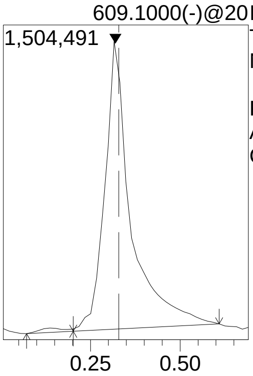

Type:Target
Name:Quercetin-3-O.hexose deoxyhex

Ret.Time:0.319
Area:6550367
Conc.:0.000ppm

623.1000(-)@21ID#:21 m/z: 623.1000

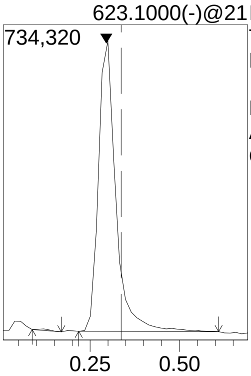

Type:Target
Name:Isorhamnetin- 3-O Rutinoside

Ret.Time:0.295
Area:2272492
Conc.:0.000ppm

447.1000(-)@22ID#:22 m/z: 447.1000

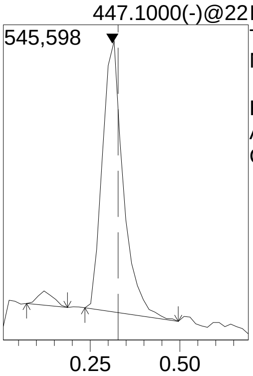

Type:Target
Name:Isorhamnetin-7-O- Pentose

Ret.Time:0.312
Area:1540703
Conc.:0.000ppm

447.1000(-)@23ID#:23 m/z: 447.1000

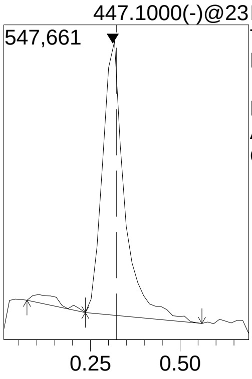

Type:Target
Name:luteoilin 7-O-glucoside

Ret.Time:0.312
Area:1657452
Conc.:0.000ppm

461.1000(-)@24ID#:24 m/z: 461.1000

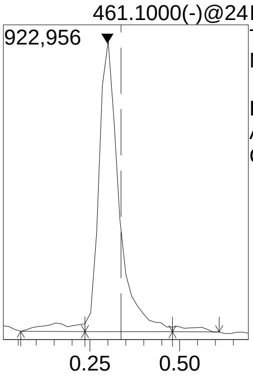

Type:Target
Name:Kaempferol-3-O-glucuronic acid

Ret.Time:0.299
Area:3114905
Conc.:0.000ppm

417.1000(-)@25ID#:25 m/z: 417.1000

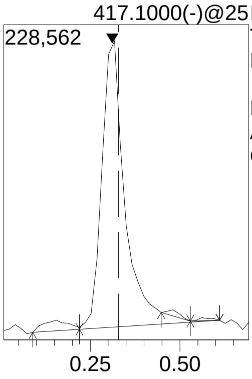

Type:Target
Name:Kaempferol-3-O-pentose

Ret.Time:0.311
Area:811284
Conc.:0.000ppm

593.1000(-)@26ID#:26 m/z: 593.1000

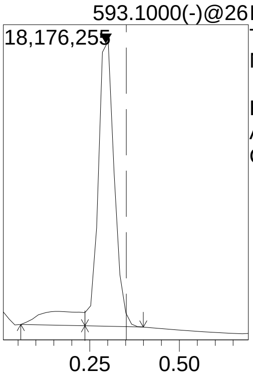

Type:Target
Name:Kaempferol-3-O-hexose deohyhe

Ret.Time:0.295
Area:49529880
Conc.:0.000ppm

153.4000(-)@27ID#:27 m/z: 153.4000

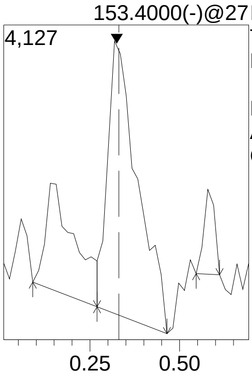

Type:Target
Name:Tyrosol

Ret.Time:0.325
Area:16877
Conc.:0.000ppm

153.0000(-)@28ID#:28 m/z: 153.0000

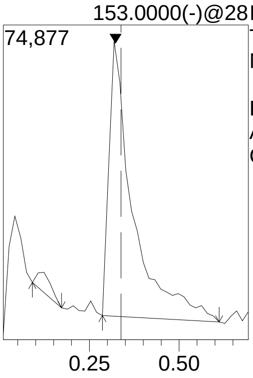

Type:Target
Name:Protocathecoic acid

Ret.Time:0.323
Area:233663
Conc.:0.000ppm

167.0000(-)@29ID#:29 m/z: 167.0000

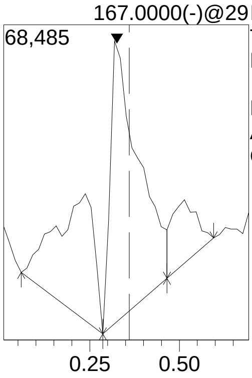

Type:Target
Name:Vanillic acid

Ret.Time:0.326
Area:227572
Conc.:0.000ppm

197.0000(-)@30ID#:30 m/z: 197.0000

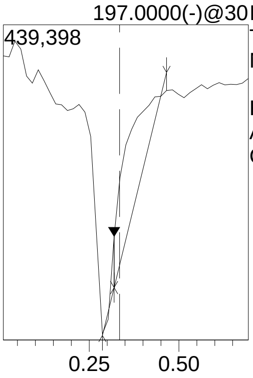

Type:Target
Name:syringic acid

Ret.Time:0.319
Area:22397
Conc.:0.000ppm

137.0000(-)@31
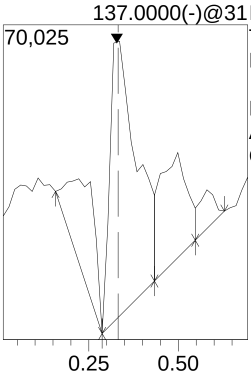

ID#:31 m/z: 137.0000
Type:Target
Name:p-hydroxybenzoic\salicilic acid

Ret.Time:0.328
Area:229438
Conc.:0.000ppm

153.0000(-)@32
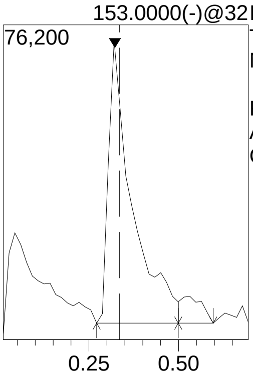

ID#:32 m/z: 153.0000
Type:Target
Name:Gentisic acid

Ret.Time:0.323
Area:237530
Conc.:0.000ppm

179.0000(-)@33
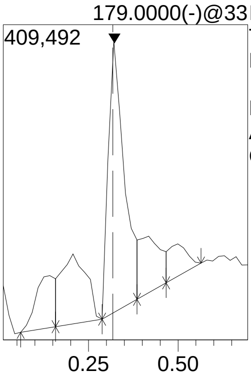

ID#:33 m/z: 179.0000
Type:Target
Name:Caffeic acid

Ret.Time:0.322
Area:785171
Conc.:0.000ppm

223.0000(-)@34
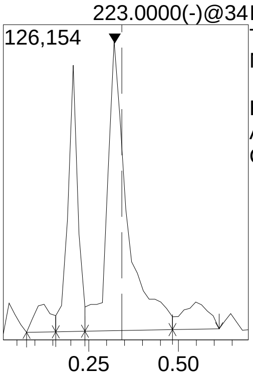

ID#:34 m/z: 223.0000
Type:Target
Name:Sinapic acid

Ret.Time:0.323
Area:328166
Conc.:0.000ppm

193.0000(-)@35
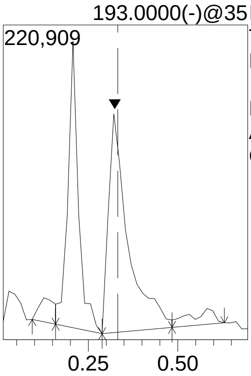

ID#:35 m/z: 193.0000
Type:Target
Name:Ferulic acid

Ret.Time:0.324
Area:408509
Conc.:0.000ppm

147.0000(-)@36
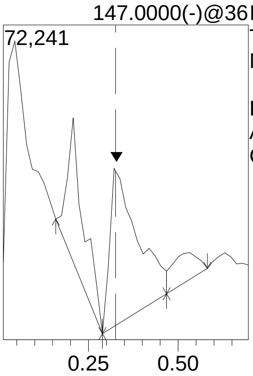

ID#:36 m/z: 147.0000
Type:Target
Name:Trans-cinnamic acid

Ret.Time:0.328
Area:161354
Conc.:0.000ppm

353.0000(-)@37
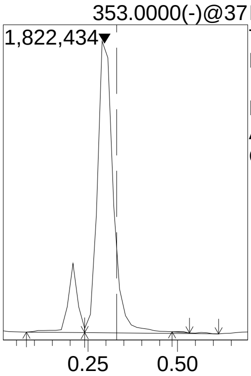

ID#:37 m/z: 353.0000
Type:Target
Name:Chlorogenic acid

Ret.Time:0.296
Area:5131151
Conc.:0.000ppm

289.0000(-)@38
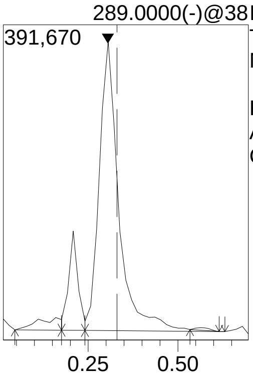

ID#:38 m/z: 289.0000
Type:Target
Name:cathechin\epicathechin

Ret.Time:0.305
Area:1247348
Conc.:0.000ppm

457.0000(-)@39ID#:39 m/z: 457.0000

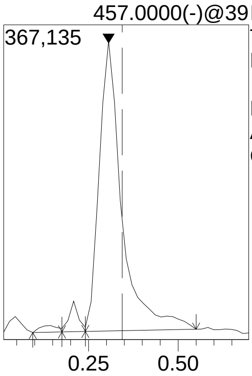

Type:Target
Name:gallocathechin\epigallocathechin

Ret.Time:0.306
Area:1344228
Conc.:0.000ppm

305.0000(-)@40ID#:40 m/z: 305.0000

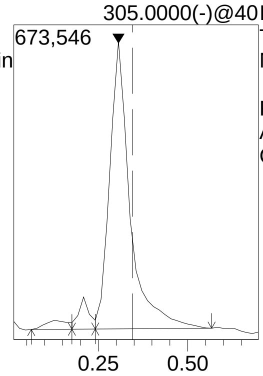

Type:Target
Name:gallocathechin\epigallocathechin

Ret.Time:0.307
Area:2274221
Conc.:0.000ppm

441.0000(-)@41ID#:41 m/z: 441.0000

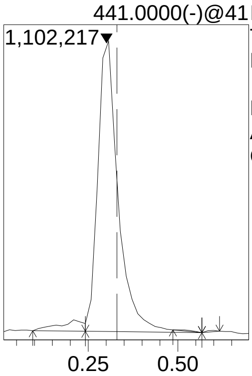

Type:Target
Name:cathechin gallate

Ret.Time:0.301
Area:3839096
Conc.:0.000ppm

577.0000(-)@42ID#:42 m/z: 577.0000

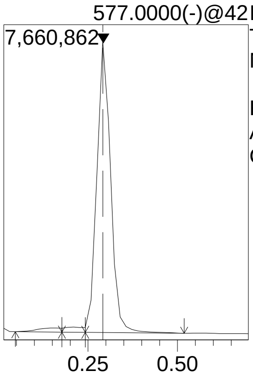

Type:Target
Name:Procianidin

Ret.Time:0.293
Area:18911219
Conc.:0.000ppm

317.0000(-)@43ID#:43 m/z: 317.0000

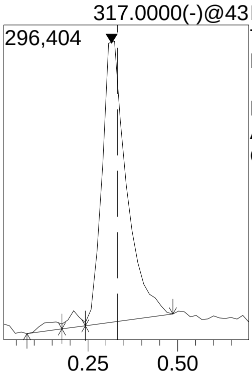

Type:Target
Name:Myricetin

Ret.Time:0.316
Area:1106867
Conc.:0.000ppm

285.0000(-)@44ID#:44 m/z: 285.0000

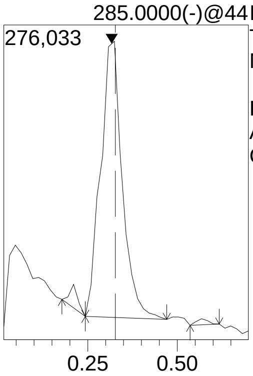

Type:Target
Name:Kaempeferol

Ret.Time:0.317
Area:833278
Conc.:0.000ppm

609.0000(-)@45ID#:45 m/z: 609.0000

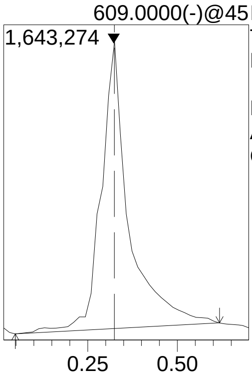

Type:Target
Name:rutin

Ret.Time:0.322
Area:7555671
Conc.:0.000ppm

579.0000(-)@46ID#:46 m/z: 579.0000

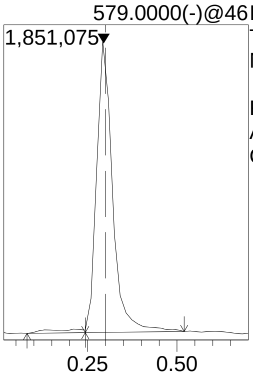

Type:Target
Name:narigin

Ret.Time:0.295
Area:5183023
Conc.:0.000ppm
